# Duodenal obstruction due to two congenital bands: a case report and literature review

**DOI:** 10.3389/fped.2025.1491520

**Published:** 2025-01-17

**Authors:** Shiqiu Xiong, Kuku Ge, Chongzhi Hou, Hongbin Yang, Hanhua Zhang, Sheng Zhang, Bailing Liu, Yuewen Hao, Ying Fang, Xiaoxia Ren

**Affiliations:** ^1^ Department of Gastroenterology, Xi’an Children’s Hospital, The Affiliated Children’s Hospital of Xi’an Jiaotong University, Xi ‘an, Shaanxi, China; ^2^ Department of General Surgery, Xi’an Children’s Hospital, The Affiliated Children’s Hospital of Xi’an Jiaotong University, Xi ‘an, Shaanxi, China; ^3^ Department of Ultrasound, Xi’an Children’s Hospital, The Affiliated Children’s Hospital of Xi’an Jiaotong University, Xi ‘an, Shaanxi, China; ^4^ Department of Radiology, Xi’an Children’s Hospital, The Affiliated Children’s Hospital of Xi’an Jiaotong University, Xi ‘an, Shaanxi, China

**Keywords:** intestinal obstruction, anomalous congenital band, clinical features, case report, literature review

## Abstract

**Introduction:**

Anomalous congenital bands are a rare cause of intestinal obstruction, with only five previously reported cases involving duodenal obstruction. We present a fifth case of duodenal obstruction due to two congenital bands and provide a comprehensive literature review summarizing the clinical features of this condition.

**Case report:**

An eight-year-old girl was admitted to our department with recurrent bilious vomiting and abdominal pain lasting six days. She had no significant past medical history, with no previous abdominal surgeries or trauma. Physical examination revealed abdominal tenderness and decreased bowel sounds. Contrast x-ray showed an obstructed passage of contrast through the third part of the duodenum. Abdominal ultrasound identified a strip-like hypoechoic structure compressing the third part of the duodenum. A diagnosis of duodenal obstruction was confirmed, and laparoscopic surgery combined with gastroduodenoscopy was performed. The procedure revealed two congenital bands adjacent to the duodenum: one extending from the duodenum to the transverse colon, and the other from the duodenum to the root of the mesentery. The bands were resected, and gastroduodenoscopy confirmed the resolution of the obstruction.

**Discussion:**

We reviewed 93 cases of anomalous congenital bands, including the present one, comprising 33 adults and 60 children, with 71.0% of the cases involving males. Common symptoms included vomiting and abdominal pain, with physical examinations often showing tenderness and distension. Imaging techniques like plain x-ray, contrast x-ray, ultrasound, and computed tomography often indicated intestinal obstruction but were less effective in directly identifying congenital bands. All cases required abdominal surgery for diagnosis and treatment. Congenital bands were primarily found attached to the ileum or its mesentery and were resected in all cases, with a favorable postoperative prognosis. This case and the literature review provide valuable insights for clinical diagnosis and treatment.

## 1 Introduction

Intestinal obstruction is a common acute emergency in children, stemming from both intrinsic and extrinsic causes (1). Typical causes include intussusception, malrotation, ileus, adhesion bands, and Hirschsprung's disease (1, 2). Anomalous congenital bands, which are neither of embryological nor inflammatory origin, are a rare cause of intestinal obstruction, with the ileum being the most commonly affected site (3). To date, over ninety cases have been reported, yet only five cases involving duodenal obstruction due to idiopathic congenital bands have been documented (4–7). This study presents an eight-year-old girl with no prior history of chronic abdominal pain, vomiting, or abdominal surgery who experienced acute duodenal obstruction due to two congenital bands. This case report has received approval from the Ethics Committee of Xi'an Children's Hospital (Approval No: 20240025). Given the rarity of congenital bands, defining their characteristics remains challenging. Therefore, we conducted a comprehensive literature review to summarize the clinical features, imaging findings, treatment strategies, and prognosis associated with intestinal obstruction due to congenital bands.

## 2 A case report

An eight-year-old girl was admitted to our department with complaints of recurrent bilious vomiting and abdominal pain persisting for six days. Her birth and family histories were unremarkable. There was no history of chronic abdominal pain, vomiting, prior abdominal surgery, or trauma. On physical examination, the patient measured 125 cm in height (25th percentile) and weighed 23 kg (25th percentile). She exhibited moderate tenderness in the upper quadrant of the abdomen, without rebound tenderness. Bowel sounds were notably reduced, with an observed frequency of two per minute.

**Figure 1:**
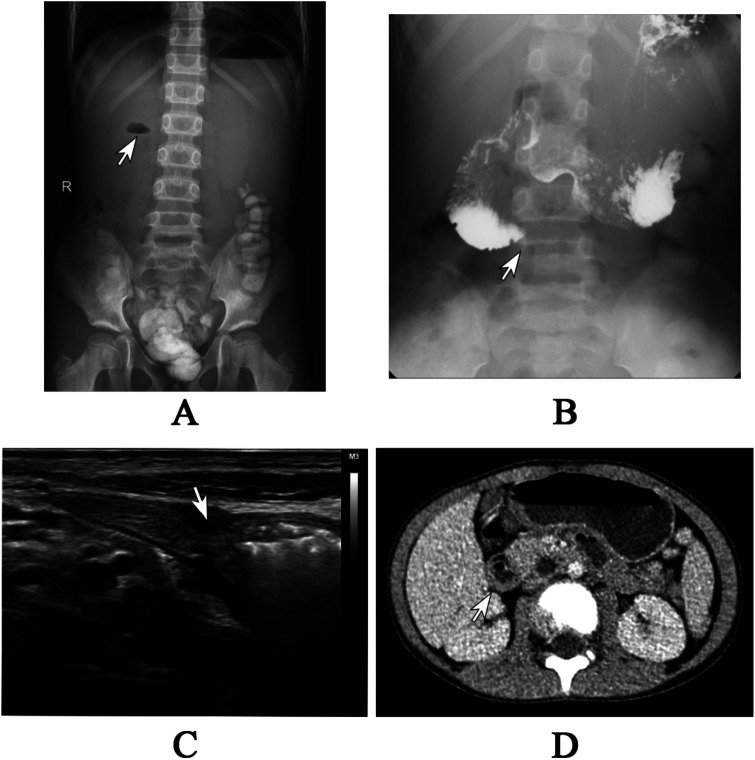
**(A)** The plain x-ray revealed a small air-fluid level in the upper right quadrant, accompanied by the absence of gas in the distal area. **(B)** The barium meal study demonstrated an obstructed passage of barium in the third segment of the duodenum. **(C)** The abdominal ultrasound showed a strip-like hypoechoic external compression affecting the third part of the duodenum, preventing the passage of contents. **(D)** The abdominal CT scan showed the distension of proximal duodenum with fluid accumulation.

**Figure 2:**
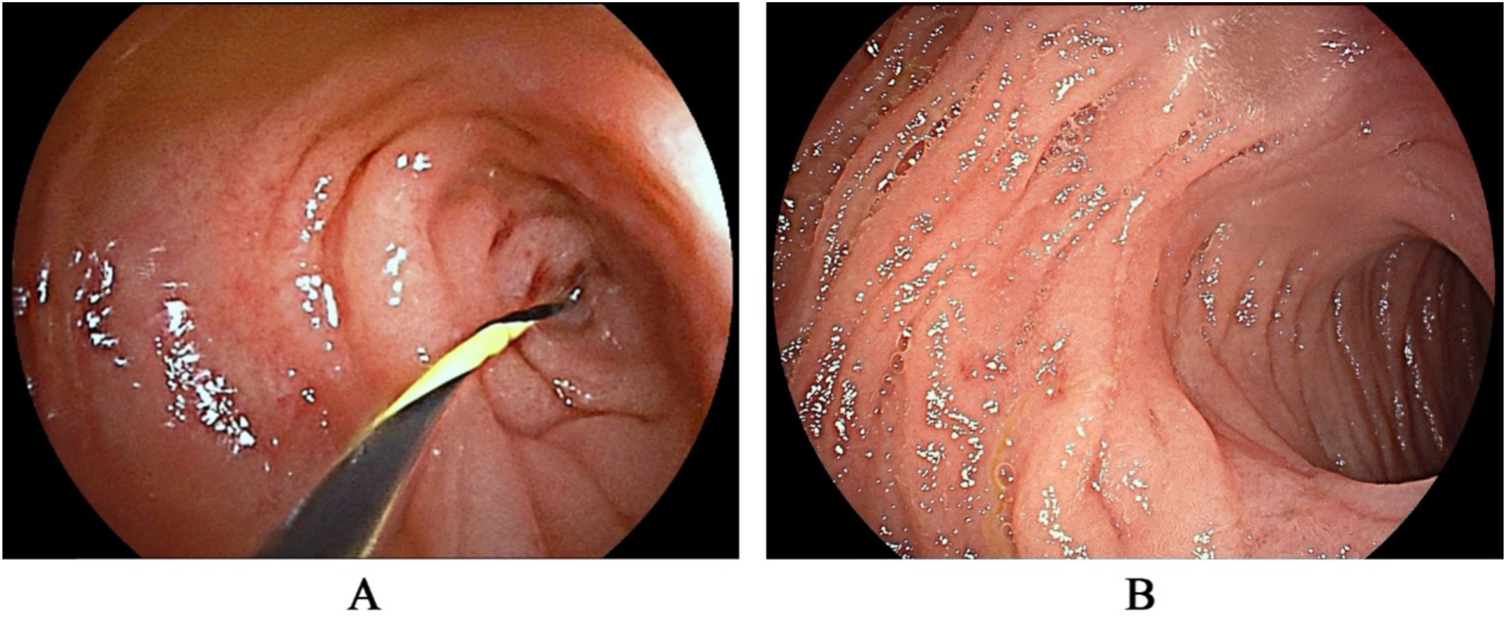
The gastroduodenoscopy revealed a distorted intestinal lumen, preventing the passage of the guidewire **(A)** after resecting the congenital bands, the distortion of the intestinal lumen was resolved **(B)**.

Laboratory data revealed an increase in serum lipase (140.4 U/L) and amylase (152 U/L), as well as elevated urine amylase (636 U/L). The plain x-ray showed a small fluid-air level with the absence of distal gas (Figure 1A). A barium meal demonstrated an obstructed passage of contrast at the third part (horizontal portion) of the duodenum (Figure 1B). Abdominal ultrasound performed after water intake revealed a strip-like hypoechoic external compression affecting the third part of the duodenum, preventing the passage of contents (Figure 1C). A contrast-enhanced computed tomography (CT) scan of the abdomen identified distension of the proximal segment of the duodenum with fluid accumulation (Figure 1D). Although the lipase and amylase were slightly increased, no evidence of pancreatitis was identified in imaging modalities. Additionally, none of the imaging studies suggested malrotation. Despite the imaging studies indicating duodenal obstruction, the exact etiology of the obstruction was not confirmed. As a result, we proceeded with gastroduodenoscopy, which revealed that the endoscope could not pass through the distorted intestinal lumen approximately 40 cm distal to the pylorus (Figure 2A).

**Figure 3:**
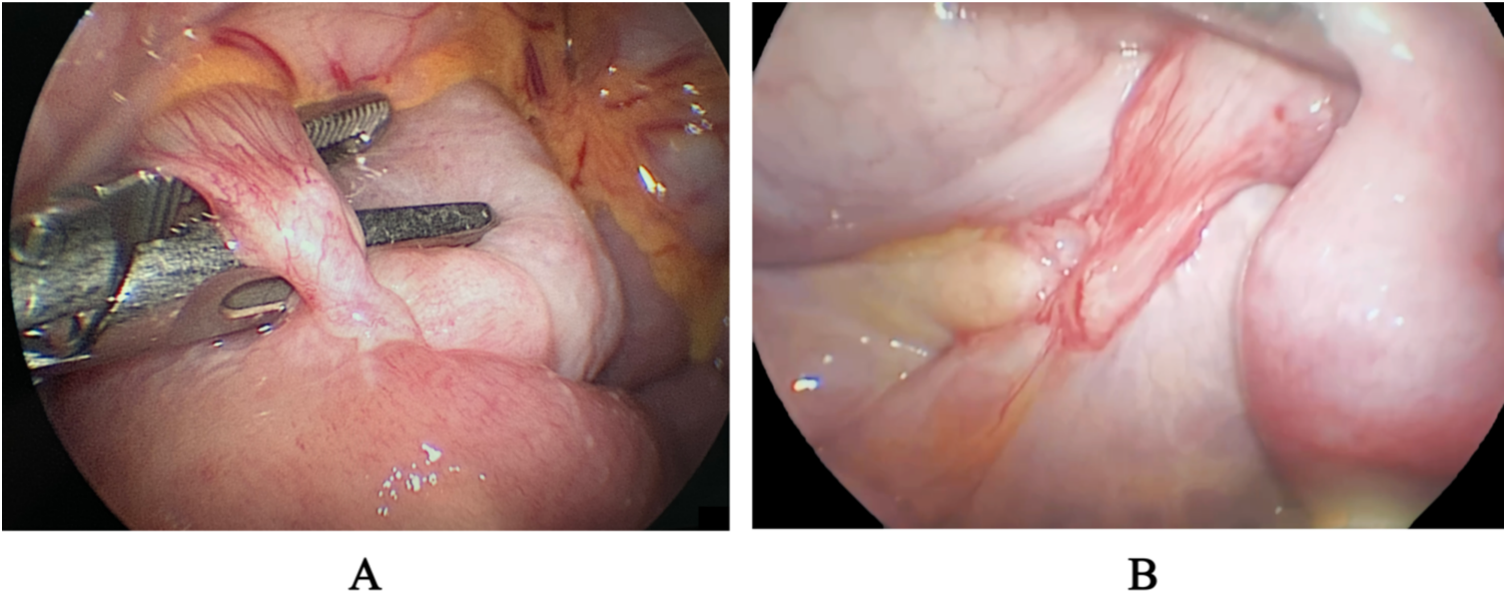
Two congenital bands were identified during the laparoscopy. One extending from the duodenum to the transverse colon, crossing over the third part of the duodenum **(A)**, and the other attached between the duodenum and the root of the mesentery **(B)**.

After conservative treatment, including fasting, gastrointestinal decompression, and nutritional support, the patient's symptoms improved, and she was able to tolerate a liquid diet. The serum lipase, amylase, and urine amylase also decreased. However, a follow-up barium meal revealed persistent obstruction at the third part of the duodenum. One week later, the patient experienced a recurrence of bilious vomiting, with 900 ml of contents. An abdominal laparoscopy combined with gastroduodenoscopy was then performed. During the procedure, two bands were identified: one extending from the duodenum to the transverse colon, crossing over the third part of the duodenum (Figure 3A), and the other attached between the duodenum and the root of the mesentery (Figure 3B). The two bands were resected, allowing contents and gas to pass freely from the duodenum to the jejunum. Additionally, a gastroduodenoscopy was conducted to confirm the resolution of the duodenal obstruction (Figure 2B). The distorted intestinal lumen was found to be unobstructed, and the endoscope passed through smoothly. The histopathological findings revealed that these congenital bands were composed of loose connective tissue.

The child had a fluid diet at the third postoperative day. She was discharged without complications on the fifth postoperative day, and subsequent outpatient follow-ups have been uneventful.

## 3 Literature review

### 3.1 Search strategy and data extraction

The PubMed database was searched for relevant literature published from the earliest available date up to August 15, 2024. We used the search terms “idiopathic congenital band*”, “congenital peritoneal band*”, “congenital band*”, “anomalous congenital band*”, and “congenital peritoneal belt*”, in titles or abstracts. To ensure thoroughness, we also searched using the terms “congenital band*”, “bowel obstruction”, and “intestinal obstruction” across all fields. Case reports and case series were included, and all references cited in the selected literature were reviewed. Only English-language articles were included. As our focus was specifically on anomalous congenital bands, we excluded literature related to Ladd's bands and the remnants of known structures, such as vitelline arteries or veins, omphalomesenteric ducts, or mesenteric remnants.

All relevant literature was meticulously reviewed, and clinical information was extracted, including demographic details, clinical features, imaging findings, therapeutic methods, and disease prognosis related to idiopathic congenital bands. The extracted data was analyzed using descriptive statistics.

### 3.2 Results

Ultimately, we included 51 papers encompassing a total of 92 cases (3–53). All patients were admitted to emergency or inpatient departments for bowel obstruction due to congenital bands. Of these cases, five involved duodenal obstruction, 67 involved small bowel obstruction, ten involved large bowel obstruction, and ten cases presented with intestinal obstruction without specification of the exact obstruction site.

#### 3.2.1 Demographics

Among the 93 cases (including the present case in our study), 33 were adults and 60 were children. Within the adult cohort, bowel obstruction primarily affected young and middle-aged adults (ages 18 to <60), comprising 78.8% (26/33) of the cases. In the pediatric group, one-third of the children (20/60) experienced bowel obstruction during infancy, with 13 cases occurring in the neonatal period (Supplementary Material I). The percentage of children affected between toddlerhood and school age was 55.0% (33/60). Only seven cases were reported in adolescents. Regarding gender distribution, the majority of patients were male, comprising 71.0% (66/93) of the cases (Supplementary Material I).

#### 3.2.2 Clinical characteristics

Clinical manifestations were detailed in 75 cases, with abdominal pain (61/75) and vomiting, either bilious or non-bilious (59/75), being the most common symptoms. Constipation was also frequently reported (Supplementary Material I). In infants with bowel obstruction, typical findings included the absence of defecation, refusal of oral intake, and abdominal distension. In terms of past medical history, eight patients had a history of chronic abdominal pain or distension, four had a history of intestinal obstruction, and 10 had undergone abdominal surgeries. The remaining patients were newly diagnosed with bowel obstruction and had no prior history of abdominal symptoms or surgeries. Physical examinations revealed abdominal distension and tenderness as the most commonly described signs. In contrast, six patients had an unremarkable abdominal examination (Supplementary Material I).

#### 3.2.3 Imaging findings

Imaging findings related to abdominal symptoms were extracted from these cases (Supplementary Material II). Of these, 15 cases underwent abdominal contrast x-ray, revealing common findings such as dilated bowels or bowel loops, with either an abrupt cutoff or restricted contrast passage. In 55 cases that utilized plain x-rays, frequent findings included dilated bowels or bowel loops, multiple air-fluid levels, and reduced bowel gas. Notably, one case involving a 16-year-old girl diagnosed with jejunal obstruction presented with a normal plain x-ray result.

Abdominal CT scan results were reported for 38 patients, typically showing dilated bowels proximal to the compression site, and collapsed bowels distal to it, with detailed documentation of compression sites, levels, and mechanisms. Notably, only two CT scans identified bands responsible for bowel obstruction. In contrast, the current case did not show a narrowing or collapsed bowel lumen on CT. Abdominal ultrasound, performed in several cases, generally demonstrated dilated bowel loops. Remarkably, the abdominal ultrasound in the present case revealed strip-like hypoechoic external compression of the duodenum.

#### 3.2.4 Abdominal surgery and prognosis

All patients underwent abdominal surgery due to a high suspicion of abdominal obstruction or the failure of conservative treatment (Supplementary Material III). Laparotomy was performed in 80 cases, while laparoscopy was utilized in 13 cases. A total of 87 congenital bands from 85 patients were meticulously documented. The origins of the bands varied, and 38/87 were found to attach to the ileum or its mesentery. Twenty-four were found to be attached to the jejunum, and 17 to the ascending colon. Other attachment sites, including the transverse colon, duodenum, Treitz ligament, and omentum, were observed in a few isolated cases. Three main mechanisms of obstruction due to congenital bands were identified: direct compression of the adjacent bowel by the band, entrapment of a bowel loop between the band and mesentery, and partial volvulus (Supplementary Material III).

All cases underwent band excision or adhesiolysis to relieve bowel obstruction. Additionally, twenty-two patients required bowel resection due to gangrenous, necrotic, non-viable, or perforated segments of the bowel. Postoperative outcomes were documented in 68 cases. Among these, three newborns succumbed to sepsis secondary to peritonitis, and one elderly female patient died from an acute coronary event on the second postoperative day. Two patients experienced intestinal obstructions due to postoperative adhesions, while the remaining patients recovered without complications.

## 4 Discussion

Based on this literature review, among 93 cases of intestinal obstruction due to congenital bands, over two-thirds were children, with the majority (53/60) experiencing their first obstruction before adolescence. Males were disproportionately affected, accounting for 71.0% of the cases. Most studies held the opinion that congenital bands were extremely rare in adults (22, 43, 44), however, we found that approximately one-third affected cases were adults. Considering their embryologic origin, congenital bands are present from birth and typically lead to clinical symptoms early in life (54). However, a portion of cases become symptomatic later in life, and the underlying reasons warrant further exploration. Similar to other congenital malformations, such as Ladd's bands or Meckel's diverticulum, congenital bands might remain asymptomatic in adults due to their specific location or structure (55–57). Occasionally, changes in the body—such as alterations in abdominal pressure, abnormal gastrointestinal motility, or age-related changes in tissue elasticity—may contribute to the onset of symptoms later in life. Moreover, it is possible that some reported “congenital bands” were actually acquired, particularly in adults, where a history of abdominal surgery, trauma, or inflammations could lead to the formation of fibrous bands that mimic congenital bands (15, 58).

Regarding clinical features, common symptoms included abdominal pain, bilious or non-bilious vomiting, and occasionally constipation, which are consistent with those seen in intestinal obstruction from other causes (59). Notably, only probably 20 patients had a history of gastrointestinal disorders, such as chronic abdominal pain, previous intestinal obstructions, or abdominal surgeries. Most patients experienced acute intestinal obstruction for the first time.

Only one study included in the literature reported elevated amylase levels (45). In our case, increased serum lipase, amylase, and urine amylase were observed on the first day of hospitalization. Following gastrointestinal decompression and abdominal laparoscopy, these parameters gradually returned to normal levels. This phenomenon is not uncommon in cases of intestinal obstruction (60, 61). Elevated lipase and amylase levels in intestinal obstruction may arise from several factors. Firstly, the distention of the duodenum during obstruction can lead to blockage of the sphincter of Oddi. Additionally, repeated vomiting may create pressure within the pancreatic duct. These two factors can contribute to the retrograde flow of pancreatic secretions through venous channels (60, 62). Furthermore, increased intestinal permeability may allow more amylase to enter the bloodstream (61, 63). These elevations do not necessarily indicate pancreatitis but are closely linked to the mechanical and physiological changes that occur during obstruction.

Image techniques such as plain x-rays, contrast x-rays, CT scans, and abdominal ultrasound demonstrated positive findings of intestinal obstruction. However, identifying the etiology was challenging. Plain x-rays might show air-fluid levels and dilated bowels, but they lacked specificity and might yield negative results at times (5, 25). Contrast x-rays could reveal dilated and narrowed bowel segments but did not provide information on the underlying cause of the obstruction. CT scans were recommended as the primary imaging modality for suspected cases of intestinal obstruction due to their ability to offer comprehensive details on diagnosis, location, level, and etiology (30, 59). However, in this review, only two cases identified congenital bands on CT, characterized by either a hyperdense (on contrast-enhanced CT) or hypodense (on non-contrast-enhanced CT) linear tissue formation (43). Ultrasound imaging also showed distended intestinal loops or signs of peritonitis, but it lacked specificity (14). In the present case, the ultrasound revealed a strip-like hypoechoic structure compressing the nearby bowel. In summary, while various imaging modalities are valuable for diagnosing intestinal obstruction, they may not always lead to a definitive preoperative diagnosis of obstruction caused by a congenital band. For this reason, surgical exploration plays a crucial role in identifying the underlying etiology.

Conservative treatment was always ineffective for managing intestinal obstruction caused by anomalous congenital bands, necessitating abdominal surgery for both diagnosis and treatment in all reviewed cases. While laparotomy has traditionally been the standard approach, laparoscopic surgery has emerged as a safe and effective alternative. Numerous case reports have demonstrated successful outcomes with laparoscopy in both pediatric (13, 25, 29, 40) and adult patients (12, 27, 30, 32), positioning it as a viable first-line surgical option. This emphasizes the importance of considering laparoscopic intervention as a standard approach for timely etiology identification and prompt treatment in cases of intestinal obstruction with an unclear cause. Predominantly, the anomalous congenital bands were identified as attaching to the ileum or its mesentery, followed by the jejunum and colon. Surgical intervention typically involved resecting these bands to alleviate bowel obstruction and, in some cases, excising non-viable or perforated bowel segments. Overall, the prognosis for intestinal obstruction was generally favorable, with most patients recovering without complications. Significantly, four patients—three newborns and one elderly individual—succumbed to severe complications following surgery, highlighting an increased risk of unfavorable outcomes in these particular patient groups.

In this literature review, we have presented a comprehensive summary of the distributions, clinical features, imaging findings, treatment strategies, and overall prognosis associated with intestinal obstruction caused by anomalous congenital bands. However, it is important to acknowledge several limitations. Firstly, our review was limited to English-language papers, potentially excluding relevant cases reported in other languages. This may have resulted in the omission of valuable data that could have provided further insights. Additionally, our review focused solely on cases documented in published databases, overlooking potentially significant cases that were unreported or not indexed. Therefore, the findings and conclusions presented in this study should be interpreted with caution, as they may not capture the full spectrum of possible scenarios.

In conclusion, we presented a rare case of duodenal obstruction caused by two anomalous congenital bands and summarized relevant cases to guide clinicians' attention toward this condition. Our findings offer valuable insights for clinical diagnosis and treatment, highlighting the need for awareness of congenital bands in similar cases.

## Data Availability

The original contributions presented in the study are included in the article/Supplementary Material, further inquiries can be directed to the corresponding authors.
