# Supplementary material for: Duodenal obstruction due to two congenital bands: a case report and literature review
Source: Front Pediatr. 2025 Jan 17;13:1491520. doi: 10.3389/fped.2025.1491520 (PMC11784611; doi:10.3389/fped.2025.1491520)
Supplement: Supplementary file 1 [file Datasheet1.pdf]

Supplementary Table 1. Clinical characteristics of patients with anomalous congenital bands

Author, year	Number	Gender	Age	Symptoms			Past medical history	Physical examination
				Vomiting	Abdominal pain	Other		
Nair, 1962(1)	1	Female	5y	✓	✓	-	A periodic vomiting associated with abdominal pain	Unremarkable
Asano, 1982(2)	2	Male	4y	✓	✓	-	No related symptoms before	On abdominal palpation, an unfixed horn-shaped tumor of clayish consistence under the umbilicus
Akgür, 1992(3)	3	Male	6d	Unclear	Unclear	-	-	Not described clearly
	4	Female	10m	Unclear	Unclear	-	No related symptoms before	-
	5	Male	3m	Unclear	Unclear	-	No related symptoms before	-
	6	Male	4y	Unclear	Unclear	-	A chronic history of abdominal pain	-
	7	Male	2.5y	Unclear	Unclear	-	A chronic history of abdominal pain	-
	8	Male	6y	Unclear	Unclear	-	A chronic history of abdominal pain	-
	9	Male	5m	Unclear	Unclear	-	No related symptoms before	-
	10	Male	9d	Unclear	Unclear	-	-	-
Lin, 1999(4)	11	Male	6m	-	-	No defecation, refuse oral intake	No related symptoms before	Unremarkable
Just, 1996(5)	12	Female	14y	✓	✓	-	-	-

Crankson, 2000(6)	13	Male	9d	✓	-	-	-	Unremarkable
Maeda, 2004(7)	14	Male	17y	-	✓	-	No related symptoms before	Abdomen distension, tenderness, and muscular resistance
Etensel, 2005(8)	15	Male	7y	✓	✓	-	Chronic mild abdomen distension and failure to thrive	Abdomen distension, tenderness, muscular resistance, and hypoactive bowel sounds
Wu, 2005(9)	16	Male	33y	✓	✓	-	No related symptoms before	Abdomen tenderness
Liu, 2005(10)	17	Male	2y	✓	-	Diarrhoea	No related symptoms before	Unremarkable
Itagaki, 2005(11)	18	Male	4.9y	✓	✓	No defecation, refuse oral intake	No related symptoms before	Abdomen tenderness and absent bowel sounds
Dimitrios, 2008(12)	19	Male	20y	✓	✓	-	No related symptoms before	Abdomen tenderness and active bowel sounds
Hunter, 2008(13)	20	Female	60y	✓	✓	-	Underwent colpopexy 20 years ago, and underwent colonoscopy for polyps several days ago	Abdomen tenderness and distension
Kumar, 2009(14)	21	Female	76y	✓	✓	Constipation	No related symptoms before	Unremarkable
Mansoor, 2009(15)	22	Female	11y	✓	✓	Constipation	No related symptoms before	Abdomen distension, tenderness, and inaudible bowel sound
Kumar, 2010(16)	23	Male	5y	✓	✓	Constipation	Underwent an operation for intestinal obstruction one year ago	Abdomen distension and hypoactive bowel sound

Fang, 2012(17)	24	Male	7y	✓	✓	Constipation	A history of constipation	Abdomen tenderness
Sarkar, 2012(18)	25	Female	8y	✓	-	-	No related symptoms before	Abdomen distension and active bowel sounds
Nouira, 2012(19)	26	Male	3y	✓	✓	-	No related symptoms before	Not described
Sozen, 2012(20)	27	Male	22y	5/10	✓	-	Two cases had bowel obstruction previously, and six had operated due to acute abdominal syndrome	Eight had abdomen distension and three had palpable mass
	28	Male	18y		✓	-		
	29	Female	33y		✓	-		
	30	Male	54y		✓	-		
	31	Male	44y		✓	-		
	32	Male	72y		✓	-		
	33	Male	38y		✓	-		
	34	Male	27y		✓	-		
	35	Male	32y		✓	-		
	36	Female	66y		✓	-		
Catania, 2013(21)	37	Male	<1w	-	-	No defecation, refuse oral intake	Underwent a caesarean section at 30 weeks' gestation with a birth weight of 900g	Abdomen distension
Low, 2013(22)	38	Male	48y	✓	✓	-	No related symptoms before	Abdomen distension
Attaallah, 2013(23)	39	Male	30y	✓	✓	-	No related symptoms before	Abdomen distension, active bowel sounds
Kostic, 2013(24)	40	Male	10y	✓	✓	Constipation	Abdominal pain after meals, and irregular passage of stool	Abdominal distension, diffuse abdominal tenderness
Sharma 2013(25)	41	Female	28y	✓	✓	Constipation	No related symptoms before	Abdominal distension, diffuse tenderness, hyperperistaltic

								bowel sounds
Leung, 2015(26)	42	Female	17d	✓	-	Irritability, decreased appetite	No related symptoms before	Abdomen distension and tenderness
Aranovich, 2015(27)	43	Female	27y	✓	✓	-	No related symptoms before	Abdomen distension
Jerraya, 2015(28)	44	Female	16y	✓	-	-	Sickle-cell anaemia	Unremarkable
Aydin, 2016(29)	45	Female	Newborn	✓	-	Abdominal distension	-	Abdomen distension
Nicolas, 2016(30)	46	Male	33y	-	✓	-	No related symptoms before	Abdomen distension and tenderness
	47	Male	18y	-	✓	-	Acute myeloblastic leukemia	Abdomen tenderness
	48	Male	19y	✓	✓	Constipation	No related symptoms before	Abdomen distension, tenderness, and hyperactive bowel sounds
Erginel, 2016(31)	49	Male	4d	✓	-	Abdominal distension	All had no related symptoms before	Not described clearly
	50	Female	1m	✓	-	Abdominal distension		
	51	Female	3y	✓	✓	-		
	52	Male	3.5y	✓	✓	-		
	53	Male	4y	✓	✓	-		
	54	Male	5y	✓	✓	-		
	55	Female	5y	✓	✓	-		

	56	Male	5y	✓	✓	-		
	57	Male	6y	✓	✓	-		
	58	Female	10y	✓	✓	-		
	59	Male	10y	✓	✓	-		
	60	Male	10y	✓	✓	-		
	61	Male	10y	✓	✓	-		
	62	Male	12y	✓	✓	-		
Wang, 2016(32)	63	Male	37y	✓	✓	-	A history of Wolff–Parkinson–White syndrome, alcohol abuse and no previous abdominal surgery	Abdomen tenderness and palpable right upper quadrant mass
Miyao, 2017(33)	64	Male	13y	✓	✓	-	A chronic history of abdominal pain	Abdomen distension, tenderness, and muscular resistance
Abdelwahed, 2017(34)	65	Male	56y	✓	✓	-	No related symptoms before	Abdomen distension and tenderness
Vishnoi, 2018(35)	66	Male	80y	✓	✓	-	Underwent a laparoscopic bilateral inguinal hernia repair; hypertension	Abdomen distension and tenderness
Cruise, 2019(36)	67	Female	20y	✓	✓	-	No related symptoms before	Abdomen tenderness
Menconi, 2019(37)	68	Male	76y	-	✓	Bowel closed to faeces and gas	Angioplasty, peptic ulcer treated with medical therapy, a discectomy following a posterior approach	Abdomen distension and tenderness
	69	Female	84y	-	✓	Bowel closed to	Atrial fibrillation	Abdomen distension

						faeces and gas		
Kerkeni, 2020(38)	70	Female	5d	Unclear	Unclear	-	Three had a chronic history of abdominal pain	Not described clearly
	71	Male	6d	Unclear	Unclear	-		
	72	Female	48d	Unclear	Unclear	-		
	73	Male	32m	Unclear	Unclear	-		
	74	Male	14m	Unclear	Unclear	-		
	75	Male	1d	Unclear	Unclear	-		
	76	Male	3d	Unclear	Unclear	-		
	77	Female	9y	Unclear	Unclear	-		
	78	Male	6y	Unclear	Unclear	-		
	79	Male	3y	Unclear	Unclear	-		
Guragai, 2020(39)	80	Male	18m	✓	-	-		
Hadded, 2021(40)	81	Female	21y	✓	✓	-	Pleural lymphoma	Abdomen tenderness
Guillen, 2021(41)	82	Male	33 d	-	-	Bilious gastric residual, abdominal distension	Underwent a caesarean section at 23 weeks gestation with a birth weight of 560g	Abdomen distension and tenderness
Parrado, 2021(42)	83	Male	17y	-	✓	Dry heaving	A history of Kawasaki disease. Underwent a laparoscopic pancreatic cyst excision at age six	Abdomen tenderness
Tepelenis,	84	Male	59y	✓	✓	-	No related symptoms before	Abdomen distension and

2021(43)								tenderness
Maree, 2022(44)	85	Male	12y	✓	✓	Constipation	No related symptoms before	Abdomen distension and tenderness
Sarraf, 2022(45)	86	Male	52y	✓	✓	Constipation	A history of hospitalization for small bowel obstruction six years ago	Abdomen distension and tenderness
Figureoa, 2022(46)	87	Male	5y	-	✓	-	Delayed speech due to impaired neurological development	Abdomen distension
Machino, 2022(47)	88	Female	67d	-	-	Bilious gastric residual, abdominal distension	Underwent a caesarean section at 28 weeks' gestation with a birth weight of 1047g	Abdomen distension
Arambepola, 2022(48)	89	Male	51y	✓	✓	Constipation	No related symptoms before	Abdomen distension
Niang, 2023(49)	90	Male	45y	✓	✓	Constipation	No related symptoms before	Abdomen distension
Naous, 2024(50)	91	Female	8d	✓	-	No defecation	-	Abdomen distension
Sleiay, 2024(51)	92	Male	29y	✓	✓	-	No related symptoms before	Abdomen distension, tenderness, and generalized rebound tenderness
Present case	93	Female	8y	✓	✓	-	No related symptoms before	Abdomen distension and tenderness

Reference

1. Nair SK, Chawla S. Congenital peritoneal band causing partial duodenal obstruction. A case report. Indian J Pediatr. 1962;29:351-4.

2. Asano S, Konuma K, Rikimaru S, Inoue K. Volvulus of the transverse colon in a four-year-old boy. Z Kinderchir. 1982;35(1):21-3.
3. Akgür FM, Tanyel FC, Büyükpamukçu N, Hiçsönmez A. Anomalous congenital bands causing intestinal obstruction in children. J Pediatr Surg. 1992;27(4):471-3.
4. Lin DS, Wang NL, Huang FY, Shih SL. Sigmoid adhesion caused by a congenital mesocolic band. J Gastroenterol. 1999;34(5):626-8.
5. Just JD, Bailey RJ. Duodenal obstruction from congenital bands: an unusual cause of pancreatitis. Can J Gastroenterol. 1996;10(7):449-50.
6. Crankson SJ, Al-Mane KA, Al-Zaben A, Al-Dhafian A. Extrinsic duodenal obstruction from anomalous congenital band. Ann Saudi Med. 2000;20(5-6):443-4.
7. Maeda A, Yokoi S, Kunou T, Tsuboi S, Niinomi N, Horisawa M, et al. Intestinal obstruction in the terminal ileum caused by an anomalous congenital vascular band between the mesoappendix and the mesentery: report of a case. Surg Today. 2004;34(9):793-5.
8. Etensel B, Ozkisacik S, Döger F, Yazici M, Gürsoy H. Anomalous congenital band: a rare cause of intestinal obstruction and failure to thrive. Pediatr Surg Int. 2005;21(12):1018-20.
9. Wu JM, Lin HF, Chen KH, Tseng LM, Huang SH. Laparoscopic diagnosis and treatment of acute small bowel obstruction resulting from a congenital band. Surg Laparosc Endosc Percutan Tech. 2005;15(5):294-6.
10. Liu C, Wu TC, Tsai HL, Chin T, Wei C. Obstruction of the proximal jejunum by an anomalous congenital band--a case report. J Pediatr Surg. 2005;40(3):E27-9.
11. Itagaki MW, Lema R, Gregory JS. Small bowel obstruction caused by a congenital jejuno-jejuno band in a child. Pediatr Emerg Care. 2005;21(10):673-4.
12. Dimitrios C, George AA, Dimosthenis Z, Nikolaos X. Intestinal obstruction due to an anomalous congenital band. Saudi J Gastroenterol. 2008;14(1):36-7.
13. Hunter IA, Sarkar R, Smith AM. Small bowel obstruction complicating colonoscopy: a case report. J Med Case Rep. 2008;2:179.
14. Kumar A, Ramakrishnan TS, Sahu S. Large Bowel Obstruction by Anomalous Congenital Band. Med J Armed Forces India. 2009;65(4):378-9.
15. Mansoor K, Al Hamidi S, Khan AM, Samujh R. Rare case of pediatric cecal volvulus. J Indian Assoc Pediatr Surg. 2009;14(3):110-2.
16. Kumar A, Ramakrishnan TS, Behl A, Sahu S, Singh G. Intestinal obstruction in a child: internal hernia caused by an anomalous congenital band. Trop Gastroenterol. 2010;31(3):219-21.

17. Fang AC, Carnell J, Stein JC. Constipation in a 7-year-old boy: congenital band causing a strangulated small bowel and pulseless electrical activity. J Emerg Med. 2012;42(3):283-7.
18. Sarkar D, Gongidi P, Presenza T, Scattergood E. Intestinal obstruction from congenital bands at the proximal jejunum: a case report and literature review. J Clin Imaging Sci. 2012;2:78.
19. Nouira F, Sarrai N, Charieg A, Jlidi S, Chaouachi B. Small bowel obstruction by an anomalous congenital band. Acta Chir Belg. 2012;112(1):77-8.
20. Sozen S, Emir S, Yazar FM, Altinsoy HK, Topuz O, Vurdem UE, et al. Small bowel obstruction due to anomalous congenital peritoneal bands - case series in adults. Bratisl Lek Listy. 2012;113(3):186-9.
21. Catania VD, Olivieri C, Nanni L, Pintus C. Extrinsic colonic obstruction by congenital fibrous band in an infant. BMJ Case Rep. 2013;2013.
22. Low SF, Ngiu CS, Sridharan R, Lee YL. Midgut malrotation with congenital peritoneal band: a rare cause of small bowel obstruction in adulthood. BMJ Case Rep. 2014;2014.
23. Attaallah W, Mokhtare S, Özden G, Yeğen C. Intestinal obstruction due to congenital mesenteric band in an adult patient. Turk J Gastroenterol. 2013;24(4):356-8.
24. Kostic A, Krstic M, Slavkovic A, Vacic N. Intestinal obstruction in children: could it be congenital abdominal bands? Pediatr Emerg Care. 2013;29(4):500-1.
25. Sharma D, Parameshwaran R, Dani T, Shetty P. Malrotation with transverse colon volvulus in early pregnancy: a rare cause for acute intestinal obstruction. BMJ Case Rep. 2013;2013.
26. Leung AA, Yamamoto J, Luca P, Beaudry P, McKeen J. Congenital Bands with Intestinal Malrotation after Propylthiouracil Exposure in Early Pregnancy. Case Rep Endocrinol. 2015;2015:789762.
27. Aranovich D, Schrier I. Reversed Intestinal Rotation Presented as Bowel Obstruction in a Pregnant Woman. Case Rep Surg. 2015;2015:870437.
28. Jerraya H, Khalfallah M, Gaja A, Dziri C. Laparoscopic treatment of intestinal obstruction caused by an uncommon congenital band. BMJ Case Rep. 2015;2015.
29. Aydin E. A rare cause of intestinal obstruction in a newborn: Congenital band compression. North Clin Istanb. 2016;3(1):75-8.
30. Nicolas G, Kfoury T, Shimlati R, Koury E, Tohmeh M, Gharios E, et al. Diagnosis and Treatment of Small Bowel Strangulation Due To Congenital Band: Three Cases of Congenital Band in Adults Lacking a History of Trauma or Surgery. Am J Case Rep. 2016;17:712-9.

31. Erginel B, Soysal FG, Ozbey H, Keskin E, Celik A, Karadag A, et al. Small Bowel Obstruction due to Anomalous Congenital Bands in Children. Gastroenterol Res Pract. 2016;2016:7364329.
32. Wang Y, Gowing S, Arena G. Adult colo-colonic intussusception caused by congenital bands: A case report and literature review. Int J Surg Case Rep. 2016;26:88-92.
33. Miyao M, Takahashi T, Uchida E. A Case of Anomalous Congenital Band that Was Difficult to Differentiate from Omphalomesenteric Duct Anomaly. J Nippon Med Sch. 2017;84(6):304-7.
34. Abdelwahed Y, Saber R, Imen BI, Hakim Z, Ayoub Z. A case report of small bowel obstruction secondary to congenital peritoneal band in adult. Int J Surg Case Rep. 2017;30:23-5.
35. Vishnoi V, Park SW, Martin P. Acute chylous peritonitis as a result of jejunal volvulus and small bowel obstruction from a congenital band adhesion. ANZ J Surg. 2019;89(7-8):E345-e6.
36. Cruise DA, Goddard K. Congenital band adhesion causing a proximal jejunal obstruction: an uncommon presentation and diagnosis. BMJ Case Rep. 2019;12(7).
37. Menconi G, Schembari E, Randazzo V, Mattone E, Coco O, Mannino M, et al. Intestinal obstruction due to congenital bands in adults who have never had abdominal surgery Two case reports and a review of the literature. Ann Ital Chir. 2019;90:524-31.
38. Kerkeni Y, Aicha B, Hamzaoui M. Idiopathic congenital anomalous bands: About ten cases with systematic review of the literature. Int J Pediatr Adolesc Med. 2020;7(4):157-60.
39. Guragai M, Bhusal S, Bhatta A. Intestinal Obstruction of Congenital Origin: A Case Report. JNMA J Nepal Med Assoc. 2020;58(221):59-61.
40. Hadded D, Mesbahi M, Zouaghi A, Marouani M, Chamekhi C, Ben Maamer A. Adult small bowel obstruction due to congenital peritoneal belt: A case report. Int J Surg Case Rep. 2021;84:106016.
41. Guillen J, Ramey S, Parimi PS. Congenital Adhesion Band Presenting as Intestinal Perforation in an Extremely Low Birth Weight Infant. AJP Rep. 2021;11(1):e1-e4.
42. Parrado RH, Rubalcava NS, Davenport KP. From the Cecum to the Sigmoid: Twisted Colon in the Pediatric Population. Cureus. 2021;13(9):e17974.
43. Tepelenis K, Stefanou SK, Stefanou CK, Tepelenis N, Margariti P, Christopoulou A, et al. Small bowel obstruction due to a congenital adhesion: a rare case report. J Surg Case Rep. 2021;2021(7):rjab282.

44. Maree G, Alelayan A, Hemi F, Shater W, Ghuzlan A, Ali W. Jejunal obstruction due to jejunocolic congenital band in a 12-year-old child: a case report. J Med Case Rep. 2022;16(1):433.
45. Sarraf K, Newman O, Mirkazemi M, Serena T. Laparoscopic Enterolysis of Congenital Band Precipitating Pathogenic Heterotopic Mesenteric Ossification Requiring Hemicolectomy: A Case Report. Am J Case Rep. 2022;23:e934910.
46. Figueroa LM, Escobar G, Osorno J, Acuña M, Solarte J. Peritonealized urachal remnant and obstructive congenital peritoneal band. A case report. Cir Pediatr. 2022;35(1):46-9.
47. Machino K, Kondo K, Sato K, Imamura T, Ohsawa Y. Strangulated bowel obstruction by idiopathic congenital band in very low birthweight infant. Pediatr Int. 2023;65(1):e15408.
48. Arambepola D, Blades H, Sinha R, Sarma D. Therapeutic emergency laparoscopy for small bowel obstruction secondary to a congenital peritoneal band. Br J Hosp Med (Lond). 2022;83(4):1-3.
49. Niang FG, Nsia RE, Faye I, Ndong A, Tendeng JN, Diedhiou M, et al. Small bowel obstruction due to congenital band in an adult: Radio-surgical correlation. Radiol Case Rep. 2024;19(1):400-2.
50. Naous A, Itani R, Itani MK, Naja Z, Rajab M. Congenital adhesion band: A rare case in a neonate. Radiol Case Rep. 2024;19(2):499-502.
51. Sleiay M, Sleiay B, Albaroudi D, Alsmoudi H, Abshi MA, Alaswad M, et al. Small bowel obstruction in a 29-year-old male with congenital peritoneal bands: A rare case report from Syria. Clin Case Rep. 2024;12(3):e8663.
