# Supplementary material for: Duodenal obstruction due to two congenital bands: a case report and literature review
Source: Front Pediatr. 2025 Jan 17;13:1491520. doi: 10.3389/fped.2025.1491520 (PMC11784611; doi:10.3389/fped.2025.1491520)
Supplement: Supplementary file 2 [file Datasheet2.pdf]

Supplementary Table 2. The imaging findings of patients

Author, year	Number	Abdominal contrast X-ray	Plain X- ray	Abdominal CT scan	Ultrasound	Other	Obstruction site
Nair, 1962(1)	1	The second part of the duodenum was dilated and persistently filled with barium. The third part of the duodenum became narrow suddenly.	-	-	-	-	Duodenal obstruction
Asano, 1982(2)	2	Barium enema revealed a transverse colon volvulus, initially blocked but later flowing through, with a 180-degree twist seen in post-evacuation imaging.	-	-	-	-	Transverse colon volvulus
Akgür, 1992(3)	3	-	Air-fluid levels were present in all cases, with free intraperitoneal air in one	-	-	-	Ileum obstruction
	4	-	-	-	-	-	Ileum obstruction
	5	-	-	-	-	-	Ileum obstruction
	6	-	-	-	-	-	Ileum obstruction
	7	-	-	-	-	-	Ileum

							obstruction
	8	-	-	-	-	-	Ileum obstruction
	9	-	-	-	-	-	Ileum obstruction
	10	-	-	-	-	-	Ascending colon obstruction
Lin, 1999(4)	11	The barium enema revealed a stenotic lesion with normal mucosa in the proximal sigmoid colon	-	-	-	-	Sigmoid colon obstruction
Just, 1996(5)	12	Duodenal spasm and partial obstruction at the fourth part of the duodenum	-	-	-	A gastroscopy showed an acute angle at the second part of her duodenum；endoscopic retrograde cholangiopancreatography (ERCP) was normal	Duodenal obstruction
Crankson, 2000(6)	13	The proximal duodenum was mildly dilated, with contrast holding up at the third duodenal part before entering the proximal jejunum.	Normal	-	-	-	Duodenal obstruction
Maeda, 2004(7)	14	-	A wide area without intestinal air	A dilated intestine in the lower abdomen and pelvic cavity	-	-	Ileum obstruction

Etensel, 2005(8)	15	-	Multiple air-fluid levels	-	-	-	Ileum obstruction
Wu, 2005(9)	16	-	Distended small bowel loops and Multiple air-fluid levels	Distended small bowel loops with thickened bowel wall and collapsed large bowel	-	-	Ileum obstruction
Liu, 2005(10)	17	Dilated third portion of the duodenum and nearly complete duodenal obstruction near the Treitz's ligament	-	-	-	-	Duodenal obstruction
Itagaki, 2005(11)	18	-	Distended small bowel loops and Multiple air-fluid levels	-	-	Endoscopy presented normal esophageal and gastric mucosa	Small bowel obstruction
Dimitrios, 2008(12)	19	-	Distended small bowel loops and Multiple air-fluid levels	-	-	-	Small bowel obstruction
Hunter, 2008(13)	20	-	Distended small bowel loop	-	-	-	Ileum obstruction
Kumar, 2009(14)	21	Dilated proximal loop of transverse and ascending colon	A few dilated small bowel loops and obverse dilated ascending and transverse colon	Progressive dilation of colon up to the distal third of transverse colon and collapsed descending colon	-	-	Transverse colon obstruction
Mansoor, 2009(15)	22	An upper GI contrast study revealed slow, progressive	Multiple air-fluid levels and a large gas-filled	-	-	-	Cecum volvulus

		contrast flow that did not fill or progress past a specific loop. A contrast enema outlined the entire colon except for the cecum.	shadow in the left abdomen				
Kumar, 2010(16)	23	-	Dilated small bowel loops	Dilated small bowel loops with stretched mesenteric vessels	-	-	Small bowel obstruction
Fang, 2012(17)	24	-	Dilated small bowel loops and multiple air-fluid levels	-	-	-	Distal ileum obstruction
Sarkar, 2012(18)	25	The proximal and descending duodenum were dilated with an abrupt cutoff in the third portion.	-	Distended duodenum with the third part tented downward; severely narrowed duodenal-jejunal junction.	Dilated proximal duodenum	-	Jejunum obstruction
Nouira, 2012(19)	26	-	Air-fluid levels	-	Normal situation of mesenteric vessels	-	Small bowel obstruction
Sozen, 2012(20)	27	-	Air-fluid levels in all patients	Progressive dilatation of small bowel segments	Dilated small bowel segments in all cases	-	Small bowel obstruction
	28	-				-	Small bowel obstruction
	29	-					Small bowel obstruction
	30	-					Small bowel

							obstruction
	31	-					Small bowel obstruction
	32	-					Small bowel obstruction
	33	-					Small bowel obstruction
	34	-					Small bowel obstruction
	35	-					Small bowel obstruction
	36	-					Small bowel obstruction
Catania, 2013(21)	37	Distended caecum	Distended bowel loops and absence of air-fluid levels	-	-	-	Ascending colon obstruction
Low, 2013(22)	38	-	Paucity of bowel gas; no dilated loops.	Dilated small bowel on the right and collapsed large bowel on the left. The superior mesenteric vein was to the left of the superior mesenteric artery	-	-	Terminal ileum obstruction
Attaallah, 2013(23)	39	-	Intestinal loops with air-fluid levels in the middle area of the abdomen	Dilation of the small bowel and free fluid in the pelvis.	-	-	Jejunal obstruction

Kostic, 2013(24)	40	-	Numerous air-fluid levels and the paucity of gas in the pelvis	-	-	-	Ileum obstruction
Sharma 2013(25)	41	-	-	-	Moderate ascites with dilated small bowel loops.	-	Transverse colon obstruction
Leung, 2015(26)	42	-	-	High-grade small bowel obstruction with multiple dilated, fluid-filled loops of bowel in the proximal ileum, characterized by muscosal hypoenhancement	-	-	Jejunum obstruction
Aranovich, 2015(27)	43	-	Distended small bowel loops in the upper abdomen and gasless lower abdomen	-	-	-	Transverse colon obstruction
Jerraya, 2015(28)	44	-	Normal	Dilation of stomach, duodenum, and proximal jejunal loops, followed by an abrupt cutoff.	-	-	Jejunal obstruction
Aydin, 2016(29)	45	No passage of contrast material beyond ileal level	Small air-fluid levels and passage of gas to distal area	-	Normal	-	Ileal obstruction
Nicolas,	46	-	-	Gastric and duodenal	-	-	Small bowel

2016(30)				distension			obstruction
	47	-	-	Distal jejunal dilation	-	-	Jejunum obstruction
	48	-	-	Small bowel dilatation of the middle third and distal third of the jejunum; a mild ileal dilation	-	-	Small bowel obstruction (jejunum and ileum)
Erginel, 2016(31)	49	-	Air-fluid levels in all patients	CT was performed in two patients and revealed dilation of the small bowel segments.	Dilated intestinal loops in all patients; partial intestinal volvulus in four patients	-	Small bowel obstruction
	50	-				-	Small bowel obstruction
	51	-				-	Small bowel obstruction
	52	-				-	Small bowel obstruction
	53	-				-	Small bowel obstruction
	54	-				-	Small bowel obstruction
	55	-				-	Small bowel obstruction
	56	-				-	Small bowel obstruction
	57	-				-	Small bowel obstruction

	58	-				-	Small bowel obstruction
	59	-				-	Small bowel obstruction
	60	-				-	Small bowel obstruction
	61	-				-	Small bowel obstruction
	62	-				-	Small bowel obstruction
Wang, 2016(32)	63	-	-	Colo-colonic intussusception with the cecum and ascending colon extending into the distal transverse colon	-	-	Colo-colonic intussusception
Miyao, 2017(33)	64	-	-	Showing a band extending from the umbilicus to the right lower quadrant of the abdomen	Little bowel gas	-	Small bowel obstruction
Abdelwahed, 2017(34)	65	-	Dilated small bowel loops with multiple air fluid levels	Dilated small bowel loops in the left side of the abdomen and collapsed loops in the right side	-	-	Ileum obstruction
Vishnoi, 2018(35)	66	-	Distended loops of small bowel	Extensive mesenteric haziness with free fluid and	-	-	Proximal jejunal obstruction

				a short segment of small bowel wall thickening			
Cruise, 2019(36)	67	Limited passage of contrast through the duodenum and into the proximal jejunum with abrupt cutoff	-	A proximal dilated jejunal loop in the left upper abdomen	-	-	Proximal jejunal obstruction
Menconi, 2019(37)	68	-	-	Mildly distended small bowel (until the distal ileum) and generalized parietal enhancement	-	-	Ileum obstruction
	69	-	-	large hernia due to rectus muscle diastasis, initially interpreted as cause of obstruction. Hernia sac filled with proximal ileum, distended, with air-fluid interface	-	-	Ileum obstruction
Kerkeni, 2020(38)	70	-	-	-	-	-	Intestinal obstruction
	71	-	-	-	-	-	Intestinal obstruction
	72	-	-	-	-	-	Intestinal obstruction
	73	-	-	-	-	-	Intestinal obstruction

	74	-	-	-	-	-	Intestinal obstruction
	75	-	-	-	-	-	Intestinal obstruction
	76	-	-	-	-	-	Intestinal obstruction
	77	-	-	-	-	-	Intestinal obstruction
	78	-	-	-	-	-	Intestinal obstruction
	79	-	-	-	-	-	Intestinal obstruction
Guragai, 2020(39)	80	-	Dilated loops of the small bowel with multiple air-fluid levels	A distended stomach and dilated duodenum, jejunum and proximal ileum loops, with collapsed colon segments	-	-	Ileum obstruction
Hadded, 2021(40)	81	-	Distended small bowel loops and multiple air-fluid levels	Distention of small bowel loops; pneumoperitoneum; peritoneal thickening around the transitional point	-	-	Small bowel obstruction
Guillen, 2021(41)	82	-	Pneumoperitoneum and dilated bowel loops	-	-	-	Jejunum obstruction
Parrado,	83	-	-	Distended and medially	-	-	Cecal volvulus

2021(42)				located cecum with fecalization of the distal ileum; decompression of the ascending colon			
Tepelenis, 2021(43)	84	-	-	Distended jejunal and ileal loops with a transition point in the right iliac fossa, beyond which the ileal loops and large bowel were collapsed.	-	-	Ileum obstruction
Maree, 2022(44)	85	Dilated stomach, duodenum, and jejunum	Dilated bowel with air-fluid levels	-	-	-	Jejunum obstruction
Sarraf, 2022(45)	86	-	-	A high-grade small bowel obstruction with a possible transition point near the terminal ileum	-	-	Terminal ileum obstruction
Figureoa, 2022(46)	87	-	Small bowel loop distension and absence of distal gas	-	-	-	Small bowel obstruction
Machino, 2022(47)	88	-	Fixed loop with intestinal dilatation	-	A dilatation swollen bowel with absent peristalsis, bowel perfusion, and free peritoneal fluid	-	Ileum obstruction

Arambepola, 2022(48)	89	-	Dilated bowel loop	The third part of the duodenum was twisted and on the right side; the stomach was distended; the dilated small bowel was lateral to the ascending colon, with twisted mesenteric vessels.	-	-	Small bowel obstruction
Niang, 2023(49)	90	-	-	Dilated small bowel loops, and a hyperdense, linear tissue formation, extending over 27 mm, and measuring 8.4 mm in thickness	-	-	Ileum obstruction
Naous, 2024(50)	91	Partial obstruction of the second part of duodenum	Double-bubble sign, distended stomach, gas in the rectum	-	-	-	Duodenal obstruction
Sleiay, 2024(51)	92	-	-	-	Dilated intestinal loops	-	Jejunum obstruction
Present case	93	Contrast passage obstructed in the horizontal part of duodenum	Several small air-fluid levels in the right upper quadrant	Proximal duodenum distension with fluid accumulation	Strip-like hypoechoic external compression of the ascending part of the duodenum and the proximal jejunum	Gastroduodenoscopy: The endoscope cannot pass through the distorted intestinal lumen 40 cm distal to the pylorus	Duodenal obstruction

Reference

1. Nair SK, Chawla S. Congenital peritoneal band causing partial duodenal obstruction. A case report. Indian J Pediatr. 1962;29:351-4.
2. Asano S, Konuma K, Rikimaru S, Inoue K. Volvulus of the transverse colon in a four-year-old boy. Z Kinderchir. 1982;35(1):21-3.
3. Akgür FM, Tanyel FC, Büyükpamukçu N, Hiçsönmez A. Anomalous congenital bands causing intestinal obstruction in children. J Pediatr Surg. 1992;27(4):471-3.
4. Lin DS, Wang NL, Huang FY, Shih SL. Sigmoid adhesion caused by a congenital mesocolic band. J Gastroenterol. 1999;34(5):626-8.
5. Just JD, Bailey RJ. Duodenal obstruction from congenital bands: an unusual cause of pancreatitis. Can J Gastroenterol. 1996;10(7):449-50.
6. Crankson SJ, Al-Mane KA, Al-Zaben A, Al-Dhafian A. Extrinsic duodenal obstruction from anomalous congenital band. Ann Saudi Med. 2000;20(5-6):443-4.
7. Maeda A, Yokoi S, Kunou T, Tsuboi S, Niinomi N, Horisawa M, et al. Intestinal obstruction in the terminal ileum caused by an anomalous congenital vascular band between the mesoappendix and the mesentery: report of a case. Surg Today. 2004;34(9):793-5.
8. Etensel B, Ozkisacik S, Döger F, Yazici M, Gürsoy H. Anomalous congenital band: a rare cause of intestinal obstruction and failure to thrive. Pediatr Surg Int. 2005;21(12):1018-20.
9. Wu JM, Lin HF, Chen KH, Tseng LM, Huang SH. Laparoscopic diagnosis and treatment of acute small bowel obstruction resulting from a congenital band. Surg Laparosc Endosc Percutan Tech. 2005;15(5):294-6.
10. Liu C, Wu TC, Tsai HL, Chin T, Wei C. Obstruction of the proximal jejunum by an anomalous congenital band--a case report. J Pediatr Surg. 2005;40(3):E27-9.
11. Itagaki MW, Lema R, Gregory JS. Small bowel obstruction caused by a congenital jejuno-jejuno band in a child. Pediatr Emerg Care. 2005;21(10):673-4.
12. Dimitrios C, George AA, Dimosthenis Z, Nikolaos X. Intestinal obstruction due to an anomalous congenital band. Saudi J Gastroenterol. 2008;14(1):36-7.
13. Hunter IA, Sarkar R, Smith AM. Small bowel obstruction complicating colonoscopy: a case report. J Med Case Rep. 2008;2:179.
14. Kumar A, Ramakrishnan TS, Sahu S. Large Bowel Obstruction by Anomalous Congenital Band. Med J Armed Forces India. 2009;65(4):378-9.

15. Mansoor K, Al Hamidi S, Khan AM, Samujh R. Rare case of pediatric cecal volvulus. J Indian Assoc Pediatr Surg. 2009;14(3):110-2.
16. Kumar A, Ramakrishnan TS, Behl A, Sahu S, Singh G. Intestinal obstruction in a child: internal hernia caused by an anomalous congenital band. Trop Gastroenterol. 2010;31(3):219-21.
17. Fang AC, Carnell J, Stein JC. Constipation in a 7-year-old boy: congenital band causing a strangulated small bowel and pulseless electrical activity. J Emerg Med. 2012;42(3):283-7.
18. Sarkar D, Gongidi P, Presenza T, Scattergood E. Intestinal obstruction from congenital bands at the proximal jejunum: a case report and literature review. J Clin Imaging Sci. 2012;2:78.
19. Nouira F, Sarrai N, Charieg A, Jlidi S, Chaouachi B. Small bowel obstruction by an anomalous congenital band. Acta Chir Belg. 2012;112(1):77-8.
20. Sozen S, Emir S, Yazar FM, Altinsoy HK, Topuz O, Vurdem UE, et al. Small bowel obstruction due to anomalous congenital peritoneal bands - case series in adults. Bratisl Lek Listy. 2012;113(3):186-9.
21. Catania VD, Olivieri C, Nanni L, Pintus C. Extrinsic colonic obstruction by congenital fibrous band in an infant. BMJ Case Rep. 2013;2013.
22. Low SF, Ngiu CS, Sridharan R, Lee YL. Midgut malrotation with congenital peritoneal band: a rare cause of small bowel obstruction in adulthood. BMJ Case Rep. 2014;2014.
23. Attaallah W, Mokhtare S, Özden G, Yeğen C. Intestinal obstruction due to congenital mesenteric band in an adult patient. Turk J Gastroenterol. 2013;24(4):356-8.
24. Kostic A, Krstic M, Slavkovic A, Vacic N. Intestinal obstruction in children: could it be congenital abdominal bands? Pediatr Emerg Care. 2013;29(4):500-1.
25. Sharma D, Parameshwaran R, Dani T, Shetty P. Malrotation with transverse colon volvulus in early pregnancy: a rare cause for acute intestinal obstruction. BMJ Case Rep. 2013;2013.
26. Leung AA, Yamamoto J, Luca P, Beaudry P, McKeen J. Congenital Bands with Intestinal Malrotation after Propylthiouracil Exposure in Early Pregnancy. Case Rep Endocrinol. 2015;2015:789762.
27. Aranovich D, Schrier I. Reversed Intestinal Rotation Presented as Bowel Obstruction in a Pregnant Woman. Case Rep Surg. 2015;2015:870437.
28. Jerraya H, Khalfallah M, Gaja A, Dziri C. Laparoscopic treatment of intestinal obstruction caused by an uncommon congenital band. BMJ Case Rep. 2015;2015.

29. Aydin E. A rare cause of intestinal obstruction in a newborn: Congenital band compression. North Clin Istanb. 2016;3(1):75-8.
30. Nicolas G, Kfoury T, Shimlati R, Koury E, Tohmeh M, Gharios E, et al. Diagnosis and Treatment of Small Bowel Strangulation Due To Congenital Band: Three Cases of Congenital Band in Adults Lacking a History of Trauma or Surgery. Am J Case Rep. 2016;17:712-9.
31. Erginel B, Soysal FG, Ozbey H, Keskin E, Celik A, Karadag A, et al. Small Bowel Obstruction due to Anomalous Congenital Bands in Children. Gastroenterol Res Pract. 2016;2016:7364329.
32. Wang Y, Gowing S, Arena G. Adult colo-colonic intussusception caused by congenital bands: A case report and literature review. Int J Surg Case Rep. 2016;26:88-92.
33. Miyao M, Takahashi T, Uchida E. A Case of Anomalous Congenital Band that Was Difficult to Differentiate from Omphalomesenteric Duct Anomaly. J Nippon Med Sch. 2017;84(6):304-7.
34. Abdelwahed Y, Saber R, Imen BI, Hakim Z, Ayoub Z. A case report of small bowel obstruction secondary to congenital peritoneal band in adult. Int J Surg Case Rep. 2017;30:23-5.
35. Vishnoi V, Park SW, Martin P. Acute chylous peritonitis as a result of jejunal volvulus and small bowel obstruction from a congenital band adhesion. ANZ J Surg. 2019;89(7-8):E345-e6.
36. Cruise DA, Goddard K. Congenital band adhesion causing a proximal jejunal obstruction: an uncommon presentation and diagnosis. BMJ Case Rep. 2019;12(7).
37. Menconi G, Schembari E, Randazzo V, Mattone E, Coco O, Mannino M, et al. Intestinal obstruction due to congenital bands in adults who have never had abdominal surgery Two case reports and a review of the literature. Ann Ital Chir. 2019;90:524-31.
38. Kerkeni Y, Aicha B, Hamzaoui M. Idiopathic congenital anomalous bands: About ten cases with systematic review of the literature. Int J Pediatr Adolesc Med. 2020;7(4):157-60.
39. Guragai M, Bhusal S, Bhatta A. Intestinal Obstruction of Congenital Origin: A Case Report. JNMA J Nepal Med Assoc. 2020;58(221):59-61.
40. Hadded D, Mesbahi M, Zouaghi A, Marouani M, Chamekhi C, Ben Maamer A. Adult small bowel obstruction due to congenital peritoneal belt: A case report. Int J Surg Case Rep. 2021;84:106016.
41. Guillen J, Ramey S, Parimi PS. Congenital Adhesion Band Presenting as Intestinal Perforation in an Extremely Low Birth Weight Infant. AJP Rep. 2021;11(1):e1-e4.
42. Parrado RH, Rubalcava NS, Davenport KP. From the Cecum to the Sigmoid: Twisted Colon in the Pediatric Population. Cureus.

2021;13(9):e17974.
43. Tepelenis K, Stefanou SK, Stefanou CK, Tepelenis N, Margariti P, Christopoulou A, et al. Small bowel obstruction due to a congenital adhesion: a rare case report. J Surg Case Rep. 2021;2021(7):rjab282.
44. Maree G, Alelayan A, Hemi F, Shater W, Ghuzlan A, Ali W. Jejunal obstruction due to jejunocolic congenital band in a 12-year-old child: a case report. J Med Case Rep. 2022;16(1):433.
45. Sarraf K, Newman O, Mirkazemi M, Serena T. Laparoscopic Enterolysis of Congenital Band Precipitating Pathogenic Heterotopic Mesenteric Ossification Requiring Hemicolectomy: A Case Report. Am J Case Rep. 2022;23:e934910.
46. Figueroa LM, Escobar G, Osorno J, Acuña M, Solarte J. Peritonealized urachal remnant and obstructive congenital peritoneal band. A case report. Cir Pediatr. 2022;35(1):46-9.
47. Machino K, Kondo K, Sato K, Imamura T, Ohsawa Y. Strangulated bowel obstruction by idiopathic congenital band in very low birthweight infant. Pediatr Int. 2023;65(1):e15408.
48. Arambepola D, Blades H, Sinha R, Sarma D. Therapeutic emergency laparoscopy for small bowel obstruction secondary to a congenital peritoneal band. Br J Hosp Med (Lond). 2022;83(4):1-3.
49. Niang FG, Nsia RE, Faye I, Ndong A, Tendeng JN, Diedhiou M, et al. Small bowel obstruction due to congenital band in an adult: Radio-surgical correlation. Radiol Case Rep. 2024;19(1):400-2.
50. Naous A, Itani R, Itani MK, Naja Z, Rajab M. Congenital adhesion band: A rare case in a neonate. Radiol Case Rep. 2024;19(2):499-502.
51. Sleiay M, Sleiay B, Albaroudi D, Alsmoudi H, Abshi MA, Alaswad M, et al. Small bowel obstruction in a 29-year-old male with congenital peritoneal bands: A rare case report from Syria. Clin Case Rep. 2024;12(3):e8663.
