# Supplementary material for: Duodenal obstruction due to two congenital bands: a case report and literature review
Source: Front Pediatr. 2025 Jan 17;13:1491520. doi: 10.3389/fped.2025.1491520 (PMC11784611; doi:10.3389/fped.2025.1491520)
Supplement: Supplementary file 3 [file Datasheet3.pdf]

Supplementary Table 3. The characteristics of anomalous congenital bands, surgery, and prognosis of patients.

Author, year	Number	Localization of congenital bands	Obstruction site	Obstruction mechanism	Surgical approach	Surgical type	Prognosis (follow-up period)
Nair, 1962(1)	1	Caecum-right upper quadrant	Duodenal obstruction	Compression	Laparotomy	Band excision	Uneventful recovery (6m)
Asano, 1982(2)	2	Transverse colon-small bowel	Transverse colon volvulus	Volvulus	Laparotomy	Band excision	Uneventful recovery (10m)
Akgür, 1992(3)	3	Ascending colon-terminal ileum	Ileum obstruction	Compression	Laparotomy	Resection and anastomosis	Uneventful recovery (1y-15y)
	4	Ascending colon-terminal ileum	Ileum obstruction	Entrapment of a loop of bowel	Laparotomy	Band excision	
	5	Ascending colon-terminal ileum mesentery	Ileum obstruction	Entrapment of a loop of bowel	Laparotomy	Resection and anastomosis	
	6	Ascending colon mesentery-terminal ileum	Ileum obstruction	Compression	Laparotomy	Band excision	
	7	Ligament of Treitz- terminal ileum mesentery	Ileum obstruction	Entrapment of a loop of bowel	Laparotomy	Band excision	
	8	Ligament of Treitz- terminal ileum mesentery	Ileum obstruction	Compression	Laparotomy	Band excision	
	9	Right lobe of liver-terminal ileum mesentery	Ileum obstruction	Compression	Laparotomy	Band excision	
	10	Right lobe of liver-ascending colon mesentery	Ascending colon	Compression	Laparotomy	Cecostomy	Died after sepsis due to peritonitis
Lin, 1999(4)	11	Left rim of the iliac fossa-sigmoid mesocolon	Sigmoid colon obstruction	Compression	Laparotomy	Band excision	Uneventful recovery (-)
Just, 1996(5)	12	-	Duodenal obstruction	Compression	Laparotomy	Band excision	Uneventful recovery (-)
Crankson,	13	Antimesenteric wall of duodenum-the root	Duodenal obstruction	Compression	Laparotomy	Band excision	Uneventful recovery

2000(6)		of the mesentery					(10m)
Maeda, 2004(7)	14	Antimesenteric side of the terminal ileum-mesoappendix	Ileum obstruction	Entrapment of a loop of bowel	Laparotomy	Band excision, intestine resection and anastomosis, and appendectomy	Uneventful recovery (15m)
Etensel, 2005(8)	15	Antimesenteric side of the ileum-sigmoid mesentery	Ileum obstruction	Compression	Laparotomy	Band excision and appendectomy	Uneventful recovery (12m)
Wu, 2005(9)	16	Greater omentum-ileum mesentery	Small bowel obstruction	Compression; entrapment of a loop of bowel	Laparoscopy	Band excision	Uneventful recovery (-)
Liu, 2005(10)	17	Antimesenteric wall of the proximal jejunum-the root of the mesentery	Duodenal obstruction	Compression	Laparotomy	Band excision	Uneventful recovery (3m)
Itagaki, 2005(11)	18	Jejunum-jejunum	Small bowel obstruction	Entrapment of a loop of bowel	Laparoscopy	Band excision	Uneventful recovery (-)
Dimitrios, 2008(12)	19	The end of the jejunum-the root of the mesentery	Small bowel obstruction	Entrapment of a loop of bowel	Laparotomy	Band excision	Uneventful recovery (-)
Hunter, 2008(13)	20	At the right iliac fossa	Ileum obstruction	Entrapment of a loop of bowel	Laparotomy	Band excision and intestinal segment resection and anastomosis	-
Kumar, 2009(14)	21	The right paravertebral region-the root of mesentery	Transverse colon obstruction	Entrapment of a loop of bowel	Laparotomy	Band excision, extended right hemicolectomy, and resection of gangrenous jejunum with jejuno-jejunostomy	Died due to acute coronary event at the second postoperative day
Mansoor, 2009(15)	22	The posterior peritoneum-the base of appendix	Cecum volvulus	Volvulus	Laparotomy	Band excision, cecum derotation, and appendicectomy	Uneventful recovery (3m)

Kumar, 2010(16)	23	The right paracolic gutter- the terminal ileum	Small bowel obstruction	Entrapment of a loop of bowel	Laparotomy	Band excision and intestinal resection	Uneventful recovery (3m)
Fang, 2012(17)	24	At the right lower quadrant of the abdomen	Distal ileum obstruction	Compression	Laparotomy	Ileum resection, ileostomy, and appendectomy	Postoperatively, the patient was managed for multiorgan failure but survived
Sarkar, 2012(18)	25	Five inches distal to the ligament of Treitz-pelvis	Jejunum obstruction	Compression	Laparotomy	Band excision	-
Nouira, 2012(19)	26	The end of the jejunum-the root of the mesentery	Small bowel obstruction	Entrapment of a loop of bowel	Laparotomy	Band excision	Uneventful recovery (24m)
Sozen, 2012(20)	27	Ascending colon-terminal ileum	Small bowel obstruction	-	Laparotomy	Band excision	Uneventful recovery (3-16m)
	28	Ascending colon-terminal ileum	Small bowel obstruction	-	Laparotomy	Band excision	
	29	Ascending colon-terminal ileum	Small bowel obstruction	-	Laparotomy	Band excision and small bowel resection	
	30	Ascending colon-terminal ileum	Small bowel obstruction	-	Laparotomy	Band excision	
	31	Jejunum-jejunum	Small bowel obstruction	-	Laparotomy	Band excision	
	32	Jejunum-jejunum	Small bowel obstruction	-	Laparotomy	Band excision	
	33	Treitz ligament-terminal ileum	Small bowel obstruction	-	Laparotomy	Band excision	
	34	Ileum to umbilicus-Meckel' diverticulum	Small bowel obstruction	-	Laparotomy	Band excision	
	35	Ileum to umbilicus-Meckel' diverticulum	Small bowel obstruction	-	Laparotomy	Band excision	
	36	-	Small bowel obstruction	-	Laparotomy	Band excision and small bowel resection	
Catania,	37	The lateral abdominal wall-duodenum-	Ascending colon	Compression	Laparotomy	Band excision	Uneventful recovery (6m)

2013(21)		jejunal flexure	obstruction				
Low, 2013(22)	38	Ileum-the distal appendix	Terminal ileum obstruction	Compression	Laparotomy	Band excision	Uneventful recovery (-)
Attaallah, 2013(23)	39	Root of the mesentery-antimesenteric wall of the jejunum	Jejunum obstruction	Compression	Laparotomy	Band excision	Uneventful recovery (-)
Kostic, 2013(24)	40	Antimesenteric wall of the jejunum-ascending colon<br>Antimesenteric wall of the jejunum-ascending colon	Ileum obstruction	Compression and Entrapment of a loop of bowel	Laparotomy	Band excision	Uneventful recovery (-)
Sharma 2013(25)	41	-	Transverse colon obstruction	Malrotation	Laparotomy	De-rotation of the colon and adhesiolysis	Uneventful recovery (-)
Leung, 2015(26)	42	-	Jejunum obstruction	Entrapment of a loop of bowel	Laparotomy	Bands ligation, bowel detorsion, and necrotic bowel resection	Uneventful recovery (-)
Aranovich, 2015(27)	43	The mesenteric root	Transverse colon obstruction	Compression	Laparotomy	Band excision	Uneventful recovery (2y)
Jerraya, 2015(28)	44	The wall of the transverse colon-the root of the mesentery	Jejunal obstruction	Entrapment of a loop of bowel	Laparoscopy	Band excision	Uneventful recovery (-)
Aydin, 2016(29)	45	-	Ileum obstruction	Compression	Laparotomy	Band excision	Uneventful recovery (-)
Nicolas, 2016(30)	46	One band at the level of the upper jejunum, the other at the angle of Treitz	Small bowel obstruction	Compression	Laparoscopy	Band excision	Uneventful recovery (-)
	47	-	Jejunum obstruction	Compression	Laparoscopy	Band excision	-
	48	One band in the mid jejunum, the other in the terminal ileum	Small bowel obstruction (jejunum and ileum)	Compression	Laparotomy	Band excision	Uneventful recovery (-)

Erginel, 2016(31)	49	Ileum-ascending colon	Small bowel obstruction	Compression in eight cases, segmental volvulus in four cases, and entrapment of an intestinal loop in four cases	Laparotomy	Band excision	-
	50	Ileum-ileum	Small bowel obstruction		Laparotomy	Band excision and ileal resection and anastomosis	-
	51	Jejunum-jejunum<br>Ileum-ileum	Small bowel obstruction		Laparotomy	Band excision	-
	52	Ascending colon-terminal ileum	Small bowel obstruction		Laparotomy	Band excision	-
	53	Ascending colon-terminal ileum	Small bowel obstruction		Laparotomy	Band excision	-
	54	Jejunum-terminal ileum	Small bowel obstruction		Laparotomy	Band excision	-
	55	Ascending colon-terminal ileum	Small bowel obstruction		Laparotomy	Band excision and ileal resection and anastomosis	-
	56	Ileum-terminal ileum	Small bowel obstruction		Laparotomy	Band excision	-
	57	Treitz-jejunum	Small bowel obstruction		Laparotomy	Band excision	-
	58	Jejunum-terminal ileum	Small bowel obstruction		Laparotomy	Band excision	-
	59	Meckel's diverticulum-ileum	Small bowel obstruction		Laparotomy	Band excision and wedge resection	-
	60	Treitz-terminal ileum	Small bowel obstruction		Laparotomy	Band excision	-
	61	Duodenum-duodenum	Small bowel obstruction		Laparotomy	Band excision	-
	62	Jejunum-terminal ileum	Small bowel obstruction		Laparotomy	Band excision	-
Wang, 2016(32)	63	In the subhepatic region	Colo-colonic intussusception	Colo-colonic intussusception	Laparoscopy	Extended right hemicolectomy with side-to-side ileocolic anastomosis	-
Miyao,	64	The mesentery of the terminal ileum-the	Small bowel obstruction	Volvulus	Laparoscopy	Band excision	-

2017(33)		umbilicus					
Abdelwahed, 2017(34)	65	Gallbladder-transverse mesocolon	Ileum obstruction	Entrapment of a loop of bowel	Laparoscopy	Band excision	Uneventful recovery (-)
Vishnoi, 2018(35)	66	Omental bands	Proximal jejunal obstruction	Volvulus	Laparotomy	Not described	-
Cruise, 2019(36)	67	Greater omentum-the antimesenteric border of a jejunal diverticulum	Proximal jejunal obstruction	-	Laparoscopy	Band excision	Uneventful recovery (2m)
Menconi, 2019(37)	68	-	Ileum obstruction	Entrapment of a loop of bowel	Laparotomy	Band excision and small intestine resection, with termino-terminal, ileo-ileal anastomosis	Uneventful recovery (-)
	69	-	Ileum obstruction	Entrapment of a loop of bowel	Laparotomy	Band excision	Uneventful recovery (-)
Kerkeni, 2020(38)	70	Antimesenteric wall of the jejunum-the root of mesentery	Intestinal obstruction	Compression in six cases, entrapment of an intestinal loop in four cases	Laparotomy	Adhesiolysis	Two died due to sepsis secondary to peritonitis, six recovered uneventfully, two had intestinal obstructions resulting from postoperative adhesion
	71	Ascending colon-duodenum	Intestinal obstruction		Laparotomy	Resection and ileostomy	
	72	Jejunum-jejunum	Intestinal obstruction		Laparotomy	Adhesiolysis	
	73	Jejunum-jejunum	Intestinal obstruction		Laparotomy	Adhesiolysis	
	74	Jejunum-jejunum	Intestinal obstruction		Laparotomy	Resection and anastomosis	
	75	Jejunum-jejunum	Intestinal obstruction		Laparotomy	Resection and ileostomy	
	76	Jejunum-jejunum	Intestinal obstruction		Laparotomy	Adhesiolysis	
	77	Antimesenteric wall of the jejunum-the root of mesentery	Intestinal obstruction		Laparotomy	Adhesiolysis	
	78	Ascending colon-terminal ileum	Intestinal obstruction		Laparotomy	Adhesiolysis	

	79	Jejunum-cecum	Intestinal obstruction		Laparotomy	Adhesiolysis	
Guragai, 2020(39)	80	35 cm proximal to the Ileocecal junction	Ileum obstruction	Malrotation	Laparotomy	Band excision	Stayed in high care unit for 11 days and discharged
Hadded, 2021(40)	81	Vesical dome-the mesentery of the ileum	Small bowel obstruction	Volvulus and entrapment of an intestinal loop	Laparotomy	Band excision, perforated small bowel resection and anastomosis	Uneventful recovery (-)
Guillen, 2021(41)	82	The base of the mesentery-the omentum	Jejunum obstruction	Volvulus and entrapment of an intestinal loop	Laparotomy	Band excision and segmental resection and anastomosis	Uneventful recovery (2m)
Parrado, 2021(42)	83	-	Cecal volvulus	Volvulus	Laparotomy	Band excision and ileocecectomy and anastomosis	Uneventful recovery (-)
Tepelenis, 2021(43)	84	The mesentery-ileum	Ileum obstruction	Entrapment of an intestinal loop	Laparotomy	Band excision	-
Maree, 2022(44)	85	The mesentery of the descending colon-the mesentery of the jejunum	Jejunum obstruction	Entrapment of an intestinal loop	Laparotomy	Band excision	Uneventful recovery (10m)
Sarraf, 2022(45)	86	-	Terminal ileum obstruction	Not described	Laparoscopy	Band excision	-
Figureoa, 2022(46)	87	Tip of the cecal appendix-mesentery	Small bowel obstruction	Compression	Laparoscopy	Band excision and urachal remnant, appendix, and Meckel’s diverticulum resection	Uneventful recovery (-)
Machino, 2022(47)	88	Ileum-the mesentery of the ileocolic vessels	Ileum obstruction	Entrapment of an intestinal loop	Laparotomy	Band excision and necrotic bowel resection and anastomosis	Uneventful recovery (5m)

Arambepola, 2022(48)	89	Anterior abdominal wall-proximal ileum	Small bowel obstruction	Rotation	Laparoscopy	Band excision	-
Niang, 2023(49)	90	The greater omentum-the anterior peritoneum	Ileum obstruction	Compression	Laparotomy	Band excision	-
Naous, 2024(50)	91	The mesentery-lateral abdominal wall	Duodenal obstruction	Compression	Laparotomy	Band excision	-
Sleiay, 2024(51)	92	Broad mesenteric origin	Jejunum obstruction	Entrapment of an intestinal loop	Laparotomy	Band excision	Uneventful recovery (-)
Present case	93	Terminal duodenum-transverse colon Duodenum-the root of the mesentery	Duodenal obstruction	Compression	Laparoscopy combined gastroduodenoscopy	Band excision	Uneventful recovery (1m)

Reference

1. Nair SK, Chawla S. Congenital peritoneal band causing partial duodenal obstruction. A case report. Indian J Pediatr. 1962;29:351-4.
2. Asano S, Konuma K, Rikimaru S, Inoue K. Volvulus of the transverse colon in a four-year-old boy. Z Kinderchir. 1982;35(1):21-3.
3. Akgür FM, Tanyel FC, Büyükpamukçu N, Hiçsönmez A. Anomalous congenital bands causing intestinal obstruction in children. J Pediatr Surg. 1992;27(4):471-3.
4. Lin DS, Wang NL, Huang FY, Shih SL. Sigmoid adhesion caused by a congenital mesocolic band. J Gastroenterol. 1999;34(5):626-8.
5. Just JD, Bailey RJ. Duodenal obstruction from congenital bands: an unusual cause of pancreatitis. Can J Gastroenterol. 1996;10(7):449-50.
6. Crankson SJ, Al-Mane KA, Al-Zaben A, Al-Dhafian A. Extrinsic duodenal obstruction from anomalous congenital band. Ann Saudi Med. 2000;20(5-6):443-4.
7. Maeda A, Yokoi S, Kunou T, Tsuboi S, Niinomi N, Horisawa M, et al. Intestinal obstruction in the terminal ileum caused by an anomalous congenital vascular band between the mesoappendix and the mesentery: report of a case. Surg Today. 2004;34(9):793-5.
8. Etensel B, Ozkisacik S, Döger F, Yazici M, Gürsoy H. Anomalous congenital band: a rare cause of intestinal obstruction and failure to thrive. Pediatr Surg Int. 2005;21(12):1018-20.
9. Wu JM, Lin HF, Chen KH, Tseng LM, Huang SH. Laparoscopic diagnosis and treatment of acute small bowel obstruction resulting from a

congenital band. Surg Laparosc Endosc Percutan Tech. 2005;15(5):294-6.
10. Liu C, Wu TC, Tsai HL, Chin T, Wei C. Obstruction of the proximal jejunum by an anomalous congenital band--a case report. J Pediatr Surg. 2005;40(3):E27-9.
11. Itagaki MW, Lema R, Gregory JS. Small bowel obstruction caused by a congenital jejuno-jejuno band in a child. Pediatr Emerg Care. 2005;21(10):673-4.
12. Dimitrios C, George AA, Dimosthenis Z, Nikolaos X. Intestinal obstruction due to an anomalous congenital band. Saudi J Gastroenterol. 2008;14(1):36-7.
13. Hunter IA, Sarkar R, Smith AM. Small bowel obstruction complicating colonoscopy: a case report. J Med Case Rep. 2008;2:179.
14. Kumar A, Ramakrishnan TS, Sahu S. Large Bowel Obstruction by Anomalous Congenital Band. Med J Armed Forces India. 2009;65(4):378-9.
15. Mansoor K, Al Hamidi S, Khan AM, Samujh R. Rare case of pediatric cecal volvulus. J Indian Assoc Pediatr Surg. 2009;14(3):110-2.
16. Kumar A, Ramakrishnan TS, Behl A, Sahu S, Singh G. Intestinal obstruction in a child: internal hernia caused by an anomalous congenital band. Trop Gastroenterol. 2010;31(3):219-21.
17. Fang AC, Carnell J, Stein JC. Constipation in a 7-year-old boy: congenital band causing a strangulated small bowel and pulseless electrical activity. J Emerg Med. 2012;42(3):283-7.
18. Sarkar D, Gongidi P, Presenza T, Scattergood E. Intestinal obstruction from congenital bands at the proximal jejunum: a case report and literature review. J Clin Imaging Sci. 2012;2:78.
19. Nouira F, Sarrai N, Charieg A, Jlidi S, Chaouachi B. Small bowel obstruction by an anomalous congenital band. Acta Chir Belg. 2012;112(1):77-8.
20. Sozen S, Emir S, Yazar FM, Altinsoy HK, Topuz O, Vurdem UE, et al. Small bowel obstruction due to anomalous congenital peritoneal bands - case series in adults. Bratisl Lek Listy. 2012;113(3):186-9.
21. Catania VD, Olivieri C, Nanni L, Pintus C. Extrinsic colonic obstruction by congenital fibrous band in an infant. BMJ Case Rep. 2013;2013.
22. Low SF, Ngiu CS, Sridharan R, Lee YL. Midgut malrotation with congenital peritoneal band: a rare cause of small bowel obstruction in adulthood. BMJ Case Rep. 2014;2014.
23. Attaallah W, Mokhtare S, Özden G, Yeğen C. Intestinal obstruction due to congenital mesenteric band in an adult patient. Turk J Gastroenterol.

2013;24(4):356-8.
24. Kostic A, Krstic M, Slavkovic A, Vacic N. Intestinal obstruction in children: could it be congenital abdominal bands? Pediatr Emerg Care. 2013;29(4):500-1.
25. Sharma D, Parameshwaran R, Dani T, Shetty P. Malrotation with transverse colon volvulus in early pregnancy: a rare cause for acute intestinal obstruction. BMJ Case Rep. 2013;2013.
26. Leung AA, Yamamoto J, Luca P, Beaudry P, McKeen J. Congenital Bands with Intestinal Malrotation after Propylthiouracil Exposure in Early Pregnancy. Case Rep Endocrinol. 2015;2015:789762.
27. Aranovich D, Schrier I. Reversed Intestinal Rotation Presented as Bowel Obstruction in a Pregnant Woman. Case Rep Surg. 2015;2015:870437.
28. Jerraya H, Khalfallah M, Gaja A, Dziri C. Laparoscopic treatment of intestinal obstruction caused by an uncommon congenital band. BMJ Case Rep. 2015;2015.
29. Aydin E. A rare cause of intestinal obstruction in a newborn: Congenital band compression. North Clin Istanb. 2016;3(1):75-8.
30. Nicolas G, Kfoury T, Shimlati R, Koury E, Tohmeh M, Gharios E, et al. Diagnosis and Treatment of Small Bowel Strangulation Due To Congenital Band: Three Cases of Congenital Band in Adults Lacking a History of Trauma or Surgery. Am J Case Rep. 2016;17:712-9.
31. Erginel B, Soysal FG, Ozbey H, Keskin E, Celik A, Karadag A, et al. Small Bowel Obstruction due to Anomalous Congenital Bands in Children. Gastroenterol Res Pract. 2016;2016:7364329.
32. Wang Y, Gowing S, Arena G. Adult colo-colonic intussusception caused by congenital bands: A case report and literature review. Int J Surg Case Rep. 2016;26:88-92.
33. Miyao M, Takahashi T, Uchida E. A Case of Anomalous Congenital Band that Was Difficult to Differentiate from Omphalomesenteric Duct Anomaly. J Nippon Med Sch. 2017;84(6):304-7.
34. Abdelwahed Y, Saber R, Imen BI, Hakim Z, Ayoub Z. A case report of small bowel obstruction secondary to congenital peritoneal band in adult. Int J Surg Case Rep. 2017;30:23-5.
35. Vishnoi V, Park SW, Martin P. Acute chylous peritonitis as a result of jejunal volvulus and small bowel obstruction from a congenital band adhesion. ANZ J Surg. 2019;89(7-8):E345-e6.
36. Cruise DA, Goddard K. Congenital band adhesion causing a proximal jejunal obstruction: an uncommon presentation and diagnosis. BMJ Case Rep. 2019;12(7).

37. Menconi G, Schembari E, Randazzo V, Mattone E, Coco O, Mannino M, et al. Intestinal obstruction due to congenital bands in adults who have never had abdominal surgery Two case reports and a review of the literature. Ann Ital Chir. 2019;90:524-31.
38. Kerkeni Y, Aicha B, Hamzaoui M. Idiopathic congenital anomalous bands: About ten cases with systematic review of the literature. Int J Pediatr Adolesc Med. 2020;7(4):157-60.
39. Guragai M, Bhusal S, Bhatta A. Intestinal Obstruction of Congenital Origin: A Case Report. JNMA J Nepal Med Assoc. 2020;58(221):59-61.
40. Hadded D, Mesbahi M, Zouaghi A, Marouani M, Chamekhi C, Ben Maamer A. Adult small bowel obstruction due to congenital peritoneal belt: A case report. Int J Surg Case Rep. 2021;84:106016.
41. Guillen J, Ramey S, Parimi PS. Congenital Adhesion Band Presenting as Intestinal Perforation in an Extremely Low Birth Weight Infant. AJP Rep. 2021;11(1):e1-e4.
42. Parrado RH, Rubalcava NS, Davenport KP. From the Cecum to the Sigmoid: Twisted Colon in the Pediatric Population. Cureus. 2021;13(9):e17974.
43. Tepelenis K, Stefanou SK, Stefanou CK, Tepelenis N, Margariti P, Christopoulou A, et al. Small bowel obstruction due to a congenital adhesion: a rare case report. J Surg Case Rep. 2021;2021(7):rjab282.
44. Maree G, Alelayan A, Hemi F, Shater W, Ghuzlan A, Ali W. Jejunal obstruction due to jejunocolic congenital band in a 12-year-old child: a case report. J Med Case Rep. 2022;16(1):433.
45. Sarraf K, Newman O, Mirkazemi M, Serena T. Laparoscopic Enterolysis of Congenital Band Precipitating Pathogenic Heterotopic Mesenteric Ossification Requiring Hemicolectomy: A Case Report. Am J Case Rep. 2022;23:e934910.
46. Figueroa LM, Escobar G, Osorno J, Acuña M, Solarte J. Peritonealized urachal remnant and obstructive congenital peritoneal band. A case report. Cir Pediatr. 2022;35(1):46-9.
47. Machino K, Kondo K, Sato K, Imamura T, Ohsawa Y. Strangulated bowel obstruction by idiopathic congenital band in very low birthweight infant. Pediatr Int. 2023;65(1):e15408.
48. Arambepola D, Blades H, Sinha R, Sarma D. Therapeutic emergency laparoscopy for small bowel obstruction secondary to a congenital peritoneal band. Br J Hosp Med (Lond). 2022;83(4):1-3.
49. Niang FG, Nsia RE, Faye I, Ndong A, Tendeng JN, Diedhiou M, et al. Small bowel obstruction due to congenital band in an adult: Radio-surgical correlation. Radiol Case Rep. 2024;19(1):400-2.

50. Naous A, Itani R, Itani MK, Naja Z, Rajab M. Congenital adhesion band: A rare case in a neonate. Radiol Case Rep. 2024;19(2):499-502.
51. Sleiay M, Sleiay B, Albaroudi D, Alsmoudi H, Abshi MA, Alaswad M, et al. Small bowel obstruction in a 29-year-old male with congenital peritoneal bands: A rare case report from Syria. Clin Case Rep. 2024;12(3):e8663.
